# The impact of functional teat number on reproductive throughput in swine

**DOI:** 10.1093/tas/txad100

**Published:** 2023-08-23

**Authors:** Dalton R Obermier, Jeremy Thomas Howard, Kent A Gray, Mark T Knauer

**Affiliations:** Department of Animal Sciences, North Carolina State University, Raleigh, NC 27607, USA; Smithfield Premium Genetics, Smithfield Foods, Smithfield, VA 23430, USA; Smithfield Premium Genetics, Smithfield Foods, Smithfield, VA 23430, USA; Department of Animal Sciences, North Carolina State University, Raleigh, NC 27607, USA

**Keywords:** litter size, piglet survival, teat number, weaning weight

## Abstract

The objective was to evaluate the impact of functional teat number on reproductive throughput in swine. Data included 735 multiparous Landrace × Large White F1 females. Sow underlined traits consisted of total teat number (**TT**), functional teat number (**FT**), nonfunctional teat number (**NFT**), and number of functional mammary glands (**FMG**). Weaning traits were calculated for both the biological and the nurse dam. For the biological dam, litter size at weaning (**LSW**) included a sow’s biological piglets regardless of cross-fostering. For nurse dam, number weaned (**NW**) included the piglets a sow weaned. For the biological dam, piglet survival (**PS**) was calculated as litter size at weaning / (total number born × 100). Linear regression estimates were calculated in RStudio v. 1.1.456 and variance components were estimated using GIBBS3F90. Average total number born, number born alive, TT, FT, NFT, and FMG were 14.22, 13.12, 14.43, 13.96, 0.42, and 10.7, respectively. An increase in one FT enhanced (*P* < 0.05) LSW by 0.32 piglets and NW by 0.33 piglets. Similarly, an increase in one FT improved (*P* < 0.05) PS by 1.63% and reduced (*P* < 0.05) preweaning mortality by 2.73%. However, an increase in one FT reduced (*P* < 0.05) average piglet weaning weight (**WW**) for biological and nurse dams by 35 and 94 g, respectively. Yet an increase in one FT enhanced (*P* < 0.05) litter weaning weight (**LWW**) for biological and nurse dams by 1.3 and 1.5 kg, respectively. Heritability estimates for TT, FT, NFT, FMG, WW, LWW, LSW, and PS were 0.25, 0.22, 0.53, 0.18, 0.21, 0.22, 0.16, and 0.18, respectively. Genetic correlation estimates between FT with TT, NFT, and FMG were 0.79, 0.09, and 0.28, respectively. Estimated genetic correlations between TT with WW, LWW, LSW, and PS were 0.37, 0.38, 0.11, and −0.19, respectively. Genetic correlation estimates between FT with WW, LWW, LSW, and PS were 0.44, 0.49, 0.39, and 0.35, respectively. Results suggest increasing functional teat number would enhance both piglet survival and reproductive throughput.

## Introduction

Piglet survival from birth to weaning continues to be a concern for the swine industry, as the percentage of piglets weaned influences both producer profitability and animal well-being. The swine industry has increased litter size at birth, yet challenges to piglet survival remain (Knauer and Hostetler, 2013). The majority of piglet mortality occurs within the first 5 d postpartum (Cabrera et al., 2012; Putz et al., 2015). Yet selection for functional teat number has perhaps not kept pace with the number of live-born piglets in hyper-prolific genetic lines (Rohrer and Nonneman, 2017). Hence, a better understanding of the associations between functional teat number, and piglet survival is warranted.

Past research (Enfield and Rempel, 1961) suggests an increase in the number of functional teats enhances the number of piglets weaned. Yet the impact of functional teat number on piglet survival should be validated in modern swine production systems. A proper understanding of sow udder morphology highlights the importance of functional teat number and helps accurately categorize teat usefulness. Functional teats must have a well-developed sphincter and produce a sufficient amount of milk. Suckling is required to initiate milk production in pigs because sows lack a mammary cistern, suggesting that one functional teat is required per piglet (Lundeheim et al., 2013). Ensuring each piglet has a functional teat at birth can reduce competition between piglets and enhance piglet survival (Speckman et al., 2021). Thus, the objective of the current study was to determine the impact of sow functional teat number on piglet survival and piglet weight at weaning.

## Materials and Methods

Data included 735 multiparous Landrace × Large White F1 females (Smithfield Premium Genetics, Rose Hill, NC), within a 2,400 head sow farm in eastern North Carolina. In gestation, sows were housed in environmentally controlled buildings on concrete slatted floors, given ad libitum access to water and fed based on sow body condition (Knauer and Baitinger, 2015). Prior to farrowing, sows were moved to individual lactation stalls where they were offered water and feed ad libitum. The lactation diet met or exceeded nutritional requirements (NRC, 2012).

Sow underline traits were quantified prior to parturition by one research technician. Prefarrow underlined traits included total teat number, functional teat number, and nonfunctional teat number. A functional teat was defined as an elongated, well-developed teat that had a predominant sphincter (Muirhead and Alexander, 1997). Nonfunctional teats included supernumerary teats, inverted teats (Muirhead and Alexander, 1997), and damaged teats that were no longer able to expel milk.

Farrowing information included room number, sow parity, total number born, number born alive, stillborn piglets, and mummified fetuses. At birth, piglets were individually tagged and weighed and reweighed at weaning. Both birth and weaning weights were collected using a Nomad Trimble scanner (Trimble Incorporated, Sunnyvale, CA), equipped with Chad 1.3 software (Smithfield Premium Genetics), attached to a Digi-Star scale (Topcon Agriculture, Livermore, CA). After individual birth weights were collected, piglets were cross-fostered to balance the number of piglets nursing each sow. All cross-fostering was performed within 48 h of farrowing. At weaning, the number of functional mammary glands was recorded by one technician when the sow was in a standing position. A functional mammary gland was classified as functional if the gland was still producing milk and had not entered mammary involution.

Weaning traits were calculated for both the biological and the nurse dam. For the biological dam, litter size at weaning included a sow’s biological piglets, regardless if the piglets had been cross-fostered to a nurse dam or not. For nurse dam weaning traits, number weaned included the piglets a sow weaned, whether they were her biological piglets or not. For the biological dam, piglet survival was calculated as litter size at weaning / (total number born × 100). For the nurse dam, preweaning mortality was computed as 100 − (number weaned  / number of piglets after cross-fostering).

Linear regression estimates were calculated using the linear model function (lm) in RStudio v. 1.1.456. Fixed effects included contemporary group (farrowing room × month) and parity category (2, 3 to 5, and 6+). For biological dam weaning traits, total number born was included as a covariate for litter size at weaning, litter weaning weight, and piglet survival. Litter size at weaning was used as a covariate for average piglet weaning weight. For wean dam production traits, number of piglets after cross-fostering was included as a covariate for average piglet weaning weight, litter weaning weight, number weaned and preweaning mortality. Number weaned was also included as a covariate in the model for average piglet weaning weight. For all weaning weight traits, piglet age was included as a covariate.

Variance components were estimated using GIBBS3F90 (Misztal et al., 2014). Both univariate and bivariate models were used for the following traits: total teat number, functional teat number, nonfunctional teat number, number of functional mammary glands, average piglet weaning weight, litter weaning weight, litter size at weaning, and piglet survival. Variance component estimation used data from biological dams. Fixed effects were identical to those used in the regression analysis. All variance component models included sow as a random effect. Sow pedigrees consisted of six generations and included the granddam, grandsire, dam, and sire. After 10,000 Gibbs samples were discarded as burn-in, 500,000 samples (saving every 20th sample) were used to calculate posterior means. Posterior means were used to compile parameter estimates and associated standard errors. Heritability estimates were calculated as the product of the additive genetic variance over the summation of the additive genetic variance and residual variance. Genetic correlations were estimated through binary analysis within POSTGIBBSF90.

## Results

Population means for reproductive traits total number born, number born alive and average piglet birth weight were 14.22, 13.12, and 1.49 kg, respectively. On average, litter size after cross-fostering was 12.78 piglets. Preweaning mortality for nurse dams was 18.0%.

Differences were observed among parity groups for underline and weaning traits. Total teat number did not differ (*P* > 0.05) between parity groups. However, parity two sows had greater (*P* < 0.01) functional teat number when compared to parity 6 + sows (14.21 vs. 13.89) and lower (*P* < 0.01) nonfunctional teat number when compared to parity 6 + sows (0.23 vs. 0.46). For production traits, parity two sows had a greater (*P* < 0.01) number of piglets after cross-fostering (13.00 vs. 12.57), number of functional mammary glands at weaning (11.21 vs. 10.21), litter size at weaning (11.00 vs. 9.90), litter weaning weight (53.3 vs. 49.8 kg) and piglet survival (78 vs. 68%) when compared to parity 6 + sows. Yet average weaning weight did not differ (*P* > 0.05) between parities.

Descriptive statistics and variance component estimates for underline traits and weaning traits are shown in Table 1. Heritability estimates for underline traits and weaning traits ranged from 0.18 to 0.53 and 0.16 to 0.22, respectively.

**Table 1.** Descriptive statistics and variance component estimates for underline traits and weaning traits of the biological dam from 735 multiparous Landrace × Large White F1 sows

Trait	Mean	σ_p_^2^	*h* ^2^	SE
Total teat number	14.43	1.14	0.25	0.04
Functional teat number	13.96	1.53	0.22	0.04
Nonfunctional teat number	0.43	0.80	0.53	0.03
Number of functional mammary glands	10.70	27.3	0.18	0.07
Average piglet weaning weight, kg	5.04	1.10	0.21	0.03
Litter weaning weight, kg	52.8	172.1	0.22	0.10
Litter size at weaning	10.16	4.42	0.16	0.08
Piglet survival, %^1^	73.2	10.43	0.18	0.08

^1^Piglet survival = litter size at weaning / (total number born × 100).

Genetic correlation estimates between the number of functional teats with total teats, nonfunctional teats, and functional mammary glands were 0.79, 0.09, and 0.28, respectively. Estimated genetic correlations between total teats with nonfunctional teats and functional mammary glands were 0.49 and 0.16, respectively. The genetic correlation estimate between nonfunctional teats and number of functional mammary glands was −0.53.

Phenotypic and genetic correlations between underline traits and weaning traits are reported in Table 2. Genetic correlations were of greater magnitude than phenotypic correlations. Functional teat number had positive genetic correlations with average piglet weaning weight, litter weaning weight, litter size at weaning, and piglet survival.

**Table 2.** Phenotypic (*r*_p_) and genetic (*r*_g_)^1^ correlations among underline and weaning traits^2^ from 735 multiparous Landrace × Large White F1 sows

	WW		LWW		LSW		PS	
Trait	*r*_p_	*r*_g_	*r*_p_	*r*_g_	*r*_p_	*r*_g_	*r*_p_	*r*_g_
Total teat number	−0.01	0.37	−0.01	0.38	0.04	0.11	0.03	−0.19
Functional teat number	−0.04	0.44	0.07	0.49	0.08	0.39	0.13	0.35
Nonfunctional teat number	0.01	0.14	−0.09	0.32	−0.11	−0.40	−0.14	−0.82
Number of functional mammary glands	0.07	0.68	0.38	0.87	0.37	0.84	0.24	0.56

^1^Calculated SE range from 0.11 to 0.56.

^2^WW, Average piglet weaning weight; LWW, litter weaning weight; LSW, litter size at weaning; PS, piglet survival = litter size at weaning / (total number born × 100).

Regression estimates associating weaning traits with underline traits are shown in Table 3. An increase in one functional teat improved (*P* < 0.05) litter size at weaning by 0.32 piglets and enhanced (*P* < 0.05) number weaned by 0.33 piglets. Similarly, an increase in one functional teat increased (*P* < 0.05) piglet survival by 1.63% and reduced (*P* < 0.05) preweaning mortality by 2.73%. An increase in functional teat number was associated (*P* < 0.05) with greater litter weaning weights for both biological dams and nurse dams. However, an increase in functional teat number was correlated (*P* < 0.05) with lighter average piglet weaning weights for both biological and nurse dams. Across all weaning traits, regression estimates for functional teat number were of greater magnitude than total teat number.

**Table 3.** Regression estimates representing production traits at weaning^1^ for underline traits from 735 multiparous Landrace × Large White F1 sows

Trait	WW, g	LWW, kg	Litter size	Survival
Biological dam				
Total teat number	−21 ± 9.0^*^	0.4 ± 0.49	0.13 ± 0.08	0.41 ± 0.56
Functional teat number	−35 ± 9.3^*^	1.3 ± 0.41^*^	0.32 ± 0.07^*^	1.63 ± 0.49^*^
Number of functional mammary glands	24 ± 13.1	2.9 ± 0.23^*^	0.52 ± 0.03^*^	6.79 ± 0.26^*^
Nurse dam				
Total teat number	−41 ± 9.1^*^	0.5 ± 0.49	0.13 ± 0.06^*^	−1.09 ± 0.56
Functional teat number	−94 ± 21.3^*^	1.5 ± 0.44^*^	0.33 ± 0.06^*^	−2.73 ± 0.59^*^
Number of functional mammary glands	13 ± 32.6	5.4 ± 0.17^*^	0.96 ± 0.01^*^	−7.47 ± 0.12^*^

^1^WW, average piglet weaning weight; LWW, litter weaning weight; litter size = Litter size at weaning (biological sow) and number weaned (nurse sow); survival = piglet survival (biological sow, Litter size at weaning / [total number born × 100]) and Preweaning mortality (nurse sow).

^*^ *P *< 0.05.

## Discussion

Reproductive throughput may be defined as the quantity of piglets or weight of piglets weaned from a cohort of sows. Knauer (2020) suggested the component traits of reproductive throughput were litter weaning weight, functional teat number, and litter size at weaning. The present study confirms substantial associations between functional teat number and reproductive throughput. An increased number of functional teats at farrowing enhanced both piglet survival and litter weaning weights. In agreement, Wiegert and Knauer (2018) reported an increase in one functional teat at farrowing improved piglet survival by 3.25% and litter weaning weight by 3.6 kg. Similarly, Speckman et al. (2021) reported an increase in one functional teat-enhanced litter size at weaning by 0.30 piglets. While Earnhardt and Knauer (2019) reported an increase in one functional teat improved number weaned by 0.26 piglets. Taken together, these current studies support past research (Enfield and Rempel, 1961; Skjervold, 1963) demonstrating the importance of functional teat number on reproductive throughput in swine systems.

The importance of underline traits increases as litter size increases relative to functional teat count. Speckman et al. (2021) divided number born alive into quartiles: Q1 ≤ 10 piglets, Q2 = 11 to 12 piglets, Q3 = 13 to 14 piglets, Q4 ≥ 15 piglets. The authors reported regression coefficients between functional teat count and litter size at weaning increased across quartiles (0.12, 0.27, 0.33, and 0.38, piglets, for Q1, Q2, Q3, and Q4, respectively). Hence as the ratio of initial litter size to functional teats increases, the number of functional teats becomes critical. This is supported by Vande Pol et al. (2021) who cross-fostered piglets, at the beginning of lactation, to fewer piglets than sow functional teat number, the same number of piglets as functional teat number or more piglets than functional teat number. The authors reported lower piglet mortality when initial litter size was less than the number of functional teats when compared to initial litter size being greater than the number of functional teats (7.7% vs. 17.9%).

The underlying biology of how increased functional teat number enhances piglet survival remains unclear. Wiegert and Knauer (2018) reported both piglet colostrum intake and sow colostrum output increased as functional teat number increased. However, in that study, average number born alive (13.1) was lower than average functional teat number (14.8). Hence, because of the litter size to functional teat ratio, colostrum quantity may not have been a limiting factor. The number of live-born piglets can be greater than the number of functional teats in hyper-prolific genetic lines (Rohrer and Nonneman, 2017). Hence, more investigation is warranted to determine if enhanced piglet survival due to increased functional teat count results from enhanced sow colostrum output, reduced competition for existing colostrum or milk, or some combination of factors.

In the present study, an increase in functional teat number was associated with lower average piglet weaning weights for both biological and nurse dams. In contrast, Wiegert and Knauer (2018) reported there was no association between functional teat number and average piglet weaning weight. Hence, the phenotypic association between functional teat number and piglet quality remains unclear.

Heritability estimates for underline traits were similar to previous literature estimates. In the current study, the heritability estimate for functional teat number (0.22) was within the range of estimates (0.17 to 0.42) reported by Chalkias et al. (2013), Lundeheim et al. (2013), and Earnhardt and Knauer (2019). For nonfunctional teat number, the heritability estimate in the current study (0.53) was greater than previous estimates (0.02 to 0.29) reported by Chalkias et al. (2013), Lundeheim et al. (2013), and Earnhardt and Knauer (2019). Perhaps differences in observed variation or technicians explain differences in heritability estimates for nonfunctional teats across studies.

In the current study, genetic correlation estimates between functional teat number with average piglet weaning weight, litter weaning weight, litter size at weaning and piglet survival ranged from 0.35 to 0.49. These genetic correlations suggest selection for an increase in functional teat number would enhance measures of reproductive throughput. This is in agreement with Earnhardt (2019) who estimated a genetic correlation between functional teat number and number weaned of 0.50. Similarly, Balzani et al. (2016) estimated a favorable genetic correlation between total teat number and a measure of piglet mortality (−0.57). In agreement, Pumfrey et al. (1980) reported a positive genetic correlation between total teat number and a measure of piglet survival (0.27). Lundeheim et al. (2013) reported genetic correlation estimates of smaller magnitude between functional teat number and litter weaning weight (0.02 to 0.13). Yet similar to the current study, Lundeheim et al. (2013) reported selection for total teat number would reduce genetic progress for weaning traits when compared to selecting for functional teat number.

In summary, the results of this study emphasize the importance of functional teat number on piglet survival and reproductive throughput. Piglet mortality is a limiting factor to enhancing animal well-being and pig farm profitability. Yet the incorporation of functional teat numbers into genetic selection programs and management techniques can help enhance piglet survival throughout the swine industry. Scientists and geneticists should use the current study to better understand associations between underline traits with weaning traits and the incorporation of genetic parameters into selection schemes.
